# Cell cycle profiling by image and flow cytometry: The optimised protocol for the detection of replicational activity using 5-Bromo-2′-deoxyuridine, low concentration of hydrochloric acid and exonuclease III

**DOI:** 10.1371/journal.pone.0175880

**Published:** 2017-04-20

**Authors:** Anna Ligasová, Petr Konečný, Ivo Frydrych, Karel Koberna

**Affiliations:** Institute of Molecular and Translational Medicine, Faculty of Medicine and Dentistry, Palacký University in Olomouc, Olomouc, Czech Republic; ENEA Centro Ricerche Casaccia, ITALY

## Abstract

The approach for the detection of replicational activity in cells using 5-bromo-2′-deoxyuridine, a low concentration of hydrochloric acid and exonuclease III is presented in the study. The described method was optimised with the aim to provide a fast and robust tool for the detection of DNA synthesis with minimal impact on the cellular structures using image and flow cytometry. The approach is based on the introduction of breaks into the DNA by the low concentration of hydrochloric acid followed by the subsequent enzymatic extension of these breaks using exonuclease III. Our data showed that the method has only a minimal effect on the tested protein localisations and is applicable both for formaldehyde- and ethanol-fixed cells. The approach partially also preserves the fluorescence of the fluorescent proteins in the HeLa cells expressing Fluorescent Ubiquitin Cell Cycle Indicator. In the case of the short labelling pulses that disabled the use of 5-ethynyl-2′-deoxyuridine because of the low specific signal, the described method provided a bright signal enabling reliable recognition of replicating cells. The optimized protocol was also successfully tested for the detection of trifluridine, the nucleoside used as an antiviral drug and in combination with tipiracil also for the treatment of some types of cancer.

## Introduction

Several methods were gradually developed for the detection of DNA synthesis in cell nuclei. Presently, the approach based on the use of 5-ethynyl-2′-deoxyuridine (EdU) and its subsequent detection by click reaction increasingly dominates [1–4]. The second widely used method is the detection of DNA replication using 5-bromo-2′-deoxyuridine (BrdU) or eventually other thymidine analogues—iodo-2′-deoxyuridine (IdU) or 5-chloro-2′-deoxyuridine (CldU)—by means of specific antibodies [5–10].

Additional methods, like the use of biotinylated nucleotides, require specific steps for their introduction. Moreover, it is not possible to control the time of their incorporation [11, 12]. The method based on the photolysis of BrdU-labelled DNA and the detection of induced breaks is another choice for the detection of DNA synthesis [13]. Although all these methods are not widely used, they can be superior to the methods based on EdU or halogen derivatives e.g. if the simultaneous detection of sensitive cellular components is required.

Both these mostly used systems have advantages and disadvantages. In the case of EdU, the most important problems are connected with its high cytotoxicity [14–16]. Therefore, EdU is not convenient for long-term studies. In addition, the click reaction by means of copper ions results in the formation of oxygen radicals, which cause at least the damage of DNA and RNA [7] and can impair the fluorescence of fluorescent proteins such as GFP as well [17]. Although the production of oxygen radicals can be minimized by the use of some additives [17], their presence can result in the lowering of the EdU signal (non-published data) and increases the costs of such an approach. On the other hand, it does not solve the relatively high EdU toxicity leading to the formation of interstrand DNA crosslinks followed by cell death [16].

Probably the most important limitation of the use of 5-halogen analogues of thymidine is that they are commonly inaccessible in the double-stranded DNA for the reaction with the specific antibodies resulting in the requirement of the use of special approaches to make them accessible. Some of these approaches are based on the use of hydrochloric acid (HCl) or sodium hydroxide, or DNase I or a mixture of nucleases or copper ions [7, 18–22]. The use of the mentioned approaches can result in the considerable changes in the cell structure including the strong and uncontrolled destruction of DNA, RNA or proteins (e.g. [7, 23]). It can also result in the loss of cells during sample preparation for the cell cycle analysis by flow cytometry. In addition, the signal depends on the antibody used [24]. Therefore, the procedures and antibodies providing a high signal/background ratio, low degradation of cellular components and high yields of cells are preferable in many situations.

In previous studies, we have shown that the incubation of fixed cells in a solution containing monovalent copper ions in the presence of oxygen results in the formation of breaks enabling the detection of BrdU by antibodies. Simultaneously, we have shown that the DNA breaks can be further extended by exonuclease activity [7]. Based on these findings, we have developed the technology for the detection of DNA synthesis in cell nuclei and also in mitochondria [7]. Although the described protocol preserves the proteins quite well and allows the detection of the whole range of cellular components, its use is not compatible with the approaches using fluorescent proteins. Another disadvantage is the necessity to prepare the reaction solution just before its use to avoid its inactivation as the reductant—the solution of sodium ascorbate—gradually oxidised in the presence of the oxygen.

In the present study, we optimised the approach of BrdU detection in nuclear DNA based on the use of HCl, which is commonly used in concentrations of 1.5–4 M [7, 25, 26]. However, these concentrations generally lead not only to the disruption of DNA structure, but also to the destruction of other cellular components [7, 23]. Our optimised approach combines the action of a low concentration of HCl (10–20 mM) and the subsequent action of exonuclease III. The described method was optimised both for formaldehyde- and ethanol-fixed cells. The method was tested also for short-time incubation with BrdU. Concurrently, we studied the impact of the approach on the detection of the chosen cellular proteins and on the detection of the fluorescent proteins in HeLa cell line expressing Fluorescent Ubiquitin Cell Cycle Indicator (FUCCI). The approach was also tested for the detection of trifluridine (TFdU)–a nucleoside analogue known as an anti-herpesvirus antiviral drug, used primarily for the treatment of eye infections caused by Herpes Simplex Virus type I [27]. Presently, it is also a component of the drug TAS-102 where it is used with combination with tipiracil for the treatment of metastatic colorectal cancer [28].

## Materials and methods

### Cell cultures, DNA labelling and fixation protocols

Human HeLa cells (cervix, adenocarcinoma; a generous gift from Dr. David Staněk, Institute of Molecular Genetics, Prague), HeLa cells stably expressing FUCCI genes (HeLa-Fucci; a generous gift from Dr. Martin Mistrík, Palacký University, Olomouc) and A549 (lung, carcinoma; ATCC) cells were used. The HeLa cells and HeLa-Fucci were cultivated in Dulbecco’s modified Eagle’s medium (DMEM, Gibco) supplemented with 10% foetal bovine serum (PAA Laboratories), 3.7 g/l of sodium bicarbonate and 50 μg/ml of gentamicin. The A549 cells were cultivated in Ham's Nutrient Mixture F12 (HyClone) medium supplemented with 10% foetal bovine serum (PAA Laboratories). The cells were cultured in the culture flasks or on coverslips (12 mm in diameter) in a Petri dish at 37°C in a humidified atmosphere containing 5% CO_2_.

For the labelling of DNA replication, BrdU was added to the culture medium for 30 minutes (if not stated otherwise). The final concentration of BrdU was 10 μM.

The cells were either fixed with 2% formaldehyde for 10 minutes, washed in 1× PBS, permeabilized in 0.2% Triton X-100 for 10 minutes and washed in 1× PBS or cells were fixed with 70% ethanol for at least 1 hr and washed with 150 mM NaCl and 3 mM KCl.

### BrdU detection

If not stated otherwise, the fixed and permeabilized cells were briefly washed with 150 mM NaCl and 3 mM KCl and subsequently incubated in 20 mM HCl for 20 minutes at 25°C. After washing by 150 mM NaCl and 3 mM KCl and 1× buffer for exonuclease III composed of 66 mM Tris-HCl, pH 8.0 and 0.66 mM MgCl_2_, samples were incubated in the solution containing 1× buffer for exonuclease III, primary anti-BrdU antibody and exonuclease III (0.4 U/μl, if not stated otherwise). In co-localization experiments, the antibodies against chosen proteins were present in the mixture with exonuclease III and anti-BrdU antibody. For the comparison of various methods of BrdU detection with respect to the signal intensity we used following protocols:

1. The fixed and permeabilised cells were washed with PBS and incubated either with 2N or 4N HCl for 20 min at 24°C. Then, the samples were washed with PBS and incubated with primary antibody raised against BrdU (clone Bu20a, diluted in PBS, 1:100, 30 min., RT) followed by their incubation with secondary antibody and DAPI [7, 24].
2. The fixed and permeabilised cells were washed with PBS and 10 mM Tris-HCl, pH 7.5 and subsequently were incubated in the solution consisting of the primary antibody, 20 U/ml DNase I and 1x buffer for DNase I (10 mM Tris-HCl; pH 7.5 at 25°C, 2.5 mM MgCl_2_, 0.1 mM CaCl_2_). The samples were incubated 30 minutes at 37°C [7, 29].
3. The fixed and permeabilized cells were washed with PBS and with a solution of 20 mM sodium ascorbate and 40 mM glycine. Then, an equal volume of 20 mM sodium ascorbate and 40 mM glycine and 8 mM CuSO4 and 200 mM NaCl was added and cells were incubated for 10 min at 300 rpm and 25°C. The samples were washed with 100 mM Tris-HCl, pH ~7.5 at 300 rpm. Then, BrdU was detected using the primary antibody supplemented with exonuclease III (1U/μl, Fermentas) in 1x buffer for exonuclease III (66 mM Tris-HCl, pH 8.0, 0.66 mM MgCl_2_) at 24°C for 30 min. Further cells were washed with 25 mM Tris-HCl, ~pH 7.5 and 150 mM NaCl and incubated with the secondary antibody and DAPI [24].

### Antibodies

These primary antibodies were used: mouse anti-BrdU antibody clones: B44 and 3D4 (BD Biosciences), Bu20a and MoBu-1 (Exbio Praha), BMC9318 (Roche) and chicken polyclonal anti-BrdU antibody (Abcam). For the concurrent localisation of DNA synthesis and cellular proteins, we used these antibodies: mouse anti-SC35 antibody (Abcam), rabbit anti-H1.2 antibody (Abcam), mouse anti-coilin antibody (Abcam), mouse anti-mitochondrial antibody (MTC02 antibody; Abcam), mouse anti-NTH1 antibody (Abcam), mouse anti-RPA32 antibody (Abcam), mouse anti-PRAF1 antibody (Abcam), rabbit anti-PCNA antibody (Abcam), and mouse anti-MCM7 antibody (Abcam).

The following secondary antibodies were used: DyLight 649 anti-mouse, Alexa Fluor^®^ 488 anti-mouse, Alexa Fluor^®^ 488 anti-rabbit and DyLight 649 anti-chicken antibodies (Jackson ImmunoResearch).

The primary antibodies were diluted either in 25 mM Tris-HCl, pH = 7.5, 150 mM NaCl or in 1× buffer for exonuclease III. The secondary antibodies were diluted in 25 mM Tris-HCl, pH = 7.5 and 150 mM NaCl. The cells were incubated with primary and secondary antibodies for 30 minutes at 37°C (if exonuclease III was used) or at room temperature (RT). After washing, the cells were mounted in the solution of 90% glycerol, 50 mM Tris-HCl, pH 8.0 and 2.5% 1,4-diazabicyclo[2.2.2]octane and evaluated.

### EdU labelling and detection

The cells were labelled with 10 μM EdU for 5 min. After fixation either with formaldehyde or ethanol, the incorporated EdU was visualised by a click reaction using Alexa Fluor 488 azide (30 min, RT, Life Sciences). The nuclear DNA was stained by DAPI (10 μM, 30 min, RT).

### Flow cytometry

A549 cells labelled with BrdU for 30 min were fixed with 70% ethanol, washed and incubated with 10 mM HCl (20 min, RT). BrdU detection was performed in a solution containing 0.4 U/μl exonuclease III, 1× buffer for exonuclease III and Bu20a primary antibody. The measurements and analyses were conducted on the flow cytometer BD FACSCalibur with 488 nm 15mW air-cooled argon-ion low-powered laser. As a fluorescence detection system, three PMT detectors (530/30; 585/42; > 670) were used. The DNA synthesis was measured and analysed using BD software CellQuest Analysis Software developed by Verity Software House, Inc. The measurements were performed for the three independent experiments. 10,000 cell nuclei were analysed per experiment.

### Fluorescence microscopy and data analysis

The images were obtained by an Olympus IX81 microscope (lenses: UPLFLN 10× NA 0.3; LUCPLFLN 20× NA 0.45; UPLANFLN 40× NA 1.3) equipped with a Hamamatsu ORCA II camera with a resolution of 1344×1024 pixels using acquisition software (Olympus).

The data were analysed using CellProfiler image analysis software [30, 31] and Microsoft Excel. The measurements were performed for the three independent experiments. For the image cytometry, 10,000 cell nuclei were analysed per experiment. The data are presented as the mean values ± SD.

The ratio between the average nuclear signal in replicating and non-replicating cells (**R/non-R**) was used to estimate the signal/noise ratio. In the case of HeLa cells, around 44 ± 6% of cells exhibited clear replicational signal after a 30-minute incubation with BrdU and optimised method of BrdU detection. It was in complete agreement with the concurrently performed analyses in which BrdU revelation was done either by 4N HCl or by monovalent copper solution or after analysis of replicating cells after a 30-minute incubation of cells with EdU. According to our results, the **R/non-R** values above 3–4 are sufficient for the clear separation of replicating and non-replicating cells (non-published results).

For the determination of the **R/non-R** ratio, we used the average nuclear signal in the (**F**–0.1) most-labelled nuclei (cells that contain the specific signal = **R**) and the signal in the (0.9–**F**) least-labelled nuclei (cells without any specific signal = **non-R**), where **F** are cells exhibiting signal after the incorporation of BrdU or EdU. For 30-minute labelling time, **F** = 0.44 (see above). In some experiments, the signal intensity was calculated. The signal intensity was equal to the average signal in the (**F**–0.1) most-labelled nuclei.

The average signal of fluorescent proteins was equal to the average nuclear signal.

## Results and discussion

### Already 5 to 10 mM HCl is sufficient for the detection of BrdU-labelled DNA if exonuclease III is used

In our previous study, we showed that the oxidative degradation of deoxyribose by copper(I) ions results in the formation of breaks enabling the detection of BrdU [7]. The signal was increased substantially after the application of exonuclease III. The use of exonuclease III allowed the significant shortening of the time of sample incubation in the cleavage solution with monovalent copper ions. In our present study, we tested the possibility to use the low concentration of HCl instead of copper(I) ions for DNA cleavage. The advantage of the system based on hydrochloric acid is the indisputable simplicity of the preparation of the solution and no special requirements for their storage.

For the optimisation of the protocol, we used mostly two anti-BrdU antibody clones: B44 and Bu20a. The affinity of B44 to BrdU exhibits only very low dependency on the BrdU position in the DNA chain and can be used in a wide range of protocols for BrdU detection. In contrast, the affinity of Bu20 antibody highly varies between different positions of BrdU in the DNA chain. It results in the high dependence of results on the BrdU detection protocol [24].

As the initial cleavage solution, 40 mM HCl was used. This concentration is usually insufficient for the detection of BrdU without additional steps. Adherent, formaldehyde-fixed cells grown on coverslip were incubated in this solution for 20 minutes at 25°C. In this respect, the commonly used HCl concentrations, enabling the successful BrdU detection at RT and incubation times around 20–30 minutes, are 1.5–4 M [7, 25, 26]. In some experiments, the incubation was performed on the laboratory shaker at 300 rpm. Subsequently, the samples were incubated with the primary antibody with exonuclease III. Concurrently, we tested the impact of the stabilization step consisting of sample incubation in formaldehyde after a short rinsing of the primary antibody. The reason for the testing of the stabilisation step is our finding that the complex of antibody with BrdU can, depending on the detection protocol and anti-BrdU antibody used, quickly dissociate resulting in the substantial lowering of the signal (non-published results). Already 0.2% formaldehyde applied shortly after the reaction with the primary anti-BrdU antibody was sufficient for effective stabilisation. Therefore, the stabilisation step was used in all other experiments, if not stated otherwise.

**Fig 1:**
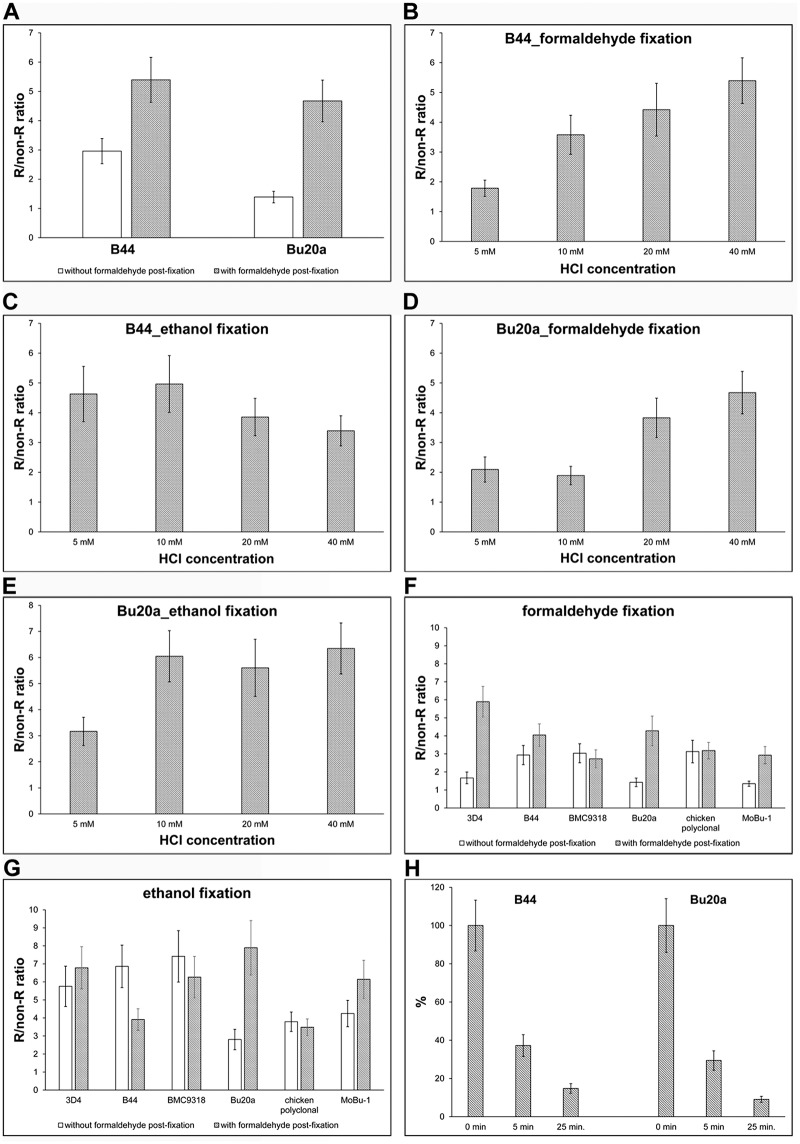
Optimising the protocol for BrdU detection. **(A)** HeLa cells were incubated with BrdU for 30 min, fixed with formaldehyde and BrdU was detected in DNA using 40 mM HCl. BrdU was detected either by the B44 or Bu20a anti-BrdU antibody with exonuclease III. The effect of formaldehyde post-fixation on the signal is shown as well. The data are presented as the mean ± SD. (**B-E)** A comparison of various HCl concentrations on BrdU signal in HeLa cells labelled for 30 min with BrdU and fixed either with formaldehyde (B, D) or ethanol (C, E). The incorporated BrdU was detected using either B44 (B, C) or Bu20a (D, E) antibody clone with exonuclease III. The data are presented as the mean ± SD. (**F, G)** A comparison of five monoclonal anti-BrdU antibody clones and one polyclonal antibody is shown. The HeLa cells were labelled with BrdU for 30 min and fixed either with formaldehyde (F) or ethanol (G). The impact of the post-fixation step is shown as well. The data are presented as the mean ± SD. (**H)** The effect of the length of the washing step on the BrdU-derived signal. The HeLa cells were labelled with BrdU for 30 min and fixed with formaldehyde. After incubation with the primary antibody, the cells were washed for 5 s (0 min) or 5 or 25 min in 1× PBS and then post-fixed with formaldehyde. The data are normalised to the % of the average signal in samples washed for 5 s in 1× PBS and then immediately post-fixed with formaldehyde. The data are presented as the mean ± SD.

It was apparent that 40 mM HCl provides the highest R/non-R ratio if we used the stabilisation step (Fig 1A). The increase was observed in the case of both antibody clones, especially Bu20a clone. Our results also showed that shaking of the cell on the laboratory shaker during incubation with 40 mM HCl had no effect on the obtained signal.

Next, we tested the different HCl concentrations with the aim of identifying the lowest concentration enabling effective BrdU detection. HeLa cells were fixed either with formaldehyde or ethanol. Ethanol fixation was selected since it is very often used in the case of sample preparation for flow cytometry analyses.

Our results showed that the lowest concentrations providing the sufficient R/non-R ratio for reliable discrimination between replicating and non-replicating cells lay between 10–40 mM in formaldehyde-fixed cells and depended on the antibody used (Fig 1B and 1D). In the case of ethanol-fixed cells, the useful HCl concentration were lower and were between 5–10 mM (Fig 1C and 1E). Concerning the comparison of anti-BrdU clones, antibody clone B44 had an R/non-R ratio of ca 4.4 in formaldehyde-fixed cells if 20 mM HCl was used and this ratio increased with the increasing HCl concentration (Fig 1B). The Bu20a clone had an R/non-R ratio of ca 3.8 and the ratio increased with the increasing HCl concentration as well (Fig 1D) When ethanol was used as the fixative, the B44 clone has the highest R/non-R ratio if 5 mM or 10 mM HCl was used (4.6 and 5.0, respectively). With the increasing HCl concentration, the R/non-R ratio was decreasing (Fig 1C). In the case of the Bu20a clone, the R/non-R ratio was ca 6 when 10 mM HCl was used and this ratio remained similar with the increasing HCl concentration (Fig 1E). In our further experiments, we used 20 mM HCl, if not stated otherwise.

We further optimised the concentration of exonuclease III. We tested concentrations in the range of 0.1 U/μl—1 U/μl. Only the lowest concentration led to a decrease of the signal. All the other tested concentrations provided a similar signal. We did not observe any difference between DAPI signals, indicating that exonuclease treatment is not accompanied by significant DNA loss. In the further experiments, a 0.4 U/μl concentration of exonuclease III was used.

According to our results, the ethanol fixation enables the use not only of a lower HCl concentration but also a lower concentration of at least some primary antibodies. In this respect, a 0.5 μg/ml concentration of B44 antibody provided a similar signal in formaldehyde-fixed cells as its four times lower concentration in ethanol-fixed cells. Interestingly, the optimal concentration of Bu20a clone was ca 2.5–5 μg/ml in both cases.

Our results also showed that the samples fixed with formaldehyde provided a better signal when the time of the washing step after formaldehyde fixation was prolonged. In this respect, we washed samples alternatively for 10, 30 minutes, 1 or 2 hrs in 25 mM Tris-HCl, pH = 7.5, 150 mM NaCl after fixation. One hour was sufficient to wash out the formaldehyde effectively. The protocols with a 1-hr washing step applied directly after fixation or after permeabilisation with Triton X-100 provided similar results. The effect of the composition of washing buffers was also compared. The following were tested 1× PBS; 25 mM Tris-HCl, pH = 7.5 and 150 mM NaCl; 0.2 M glycine in 1× PBS; 0.2 M glycine in 25 mM Tris-HCl, pH = 7.5 and 150 mM NaCl or 0.2 M glycine in 100 mM Tris-HCl, pH = 7.5. No significant differences between washing buffers was observed. In the case of ethanol fixation, such intensive washing was not necessary.

We also compared the R/non-R ratio of various methods of BrdU detection. In these experiments, cells were incubated with 10 μM BrdU for 30 min and the incorporated BrdU was detected using five approaches based on: 1) 2N; 2) 4N HCl; 3) 20 mM HCl and exonuclease III; 4) monovalent copper ions and exonuclease III and 5) DNase I. It was obvious that the protocol based on the use of 20 mM HCl and exonuclease III treatment provided the highest R/non-R ratio (S1 Fig).

### The different anti-BrdU antibody clones provide different signal intensity

In the next experiments, we compared six different anti-BrdU antibodies in HeLa cells labelled for 30 min with BrdU and fixed either with formaldehyde or ethanol (Fig 1F and 1G). Within these experiments, we also compared how the stabilisation step influenced the signal strength in HCl- and exonuclease III-treated cells. It is obvious that in the formaldehyde- fixed cells, the highest specific signal was provided by the antibody clones 3D4, B44 and Bu20a if the stabilisation of the antibody was used. These clones provided a sufficient signal and had only a minimal background (Fig 1F). Only two antibodies, BMC9318 monoclonal antibody and polyclonal antibody, did not show any impact of the stabilisation step on the signal strength.

In the case of the ethanol-fixated cells and stabilisation step, the highest R/non-R ratio and therefore the specific signal was provided by the clone Bu20a. On the other hand, the signal of this antibody significantly decreased without stabilisation (Fig 1G). The situation was completely different if B44 and BMC9318 clones were used. The stabilisation step resulted in an increase of the background and consequently to a lowering of the R/non-R ratio. Similarly, the polyclonal antibody was independent on the stabilisation. In this case, the signal was almost the same, independently of whether stabilisation was used or not (Fig 1G).

It is clear from the obtained data that if ethanol-fixed cells are used for the determination of replicational activity using BrdU, the most suitable monoclonal antibody clone is Bu20a due to the extremely high R/non-R ratio, although it needs the stabilisation step. It is also obvious that the stabilisation step plays a more important role if formaldehyde-fixed cells are used.

Further, we analysed the decrease of the signal after the variable-length incubation time in the washing buffer (25 mM Tris-HCl, pH = 7.5, 150 mM NaCl) before the stabilisation step (Fig 1H). In this case, the samples were fixed with formaldehyde after incubation with exonuclease III and anti-BrdU antibody, washed in PBS for ten seconds (0 min in the graph in Fig 1H) or for 5 or 25 minutes and stabilised with 0.2% formaldehyde. The signal intensity was inversely proportional to the time of the incubation in the washing buffer (Fig 1H). This result was in complete agreement with our non-published data showing that some antibodies form very unstable complexes with BrdU in the case of some BrdU detection protocols and that these complexes can be stabilised by a low concentration of formaldehyde.

### The impact of the optimised method on protein detection including the fluorescent proteins

**Fig 2:**
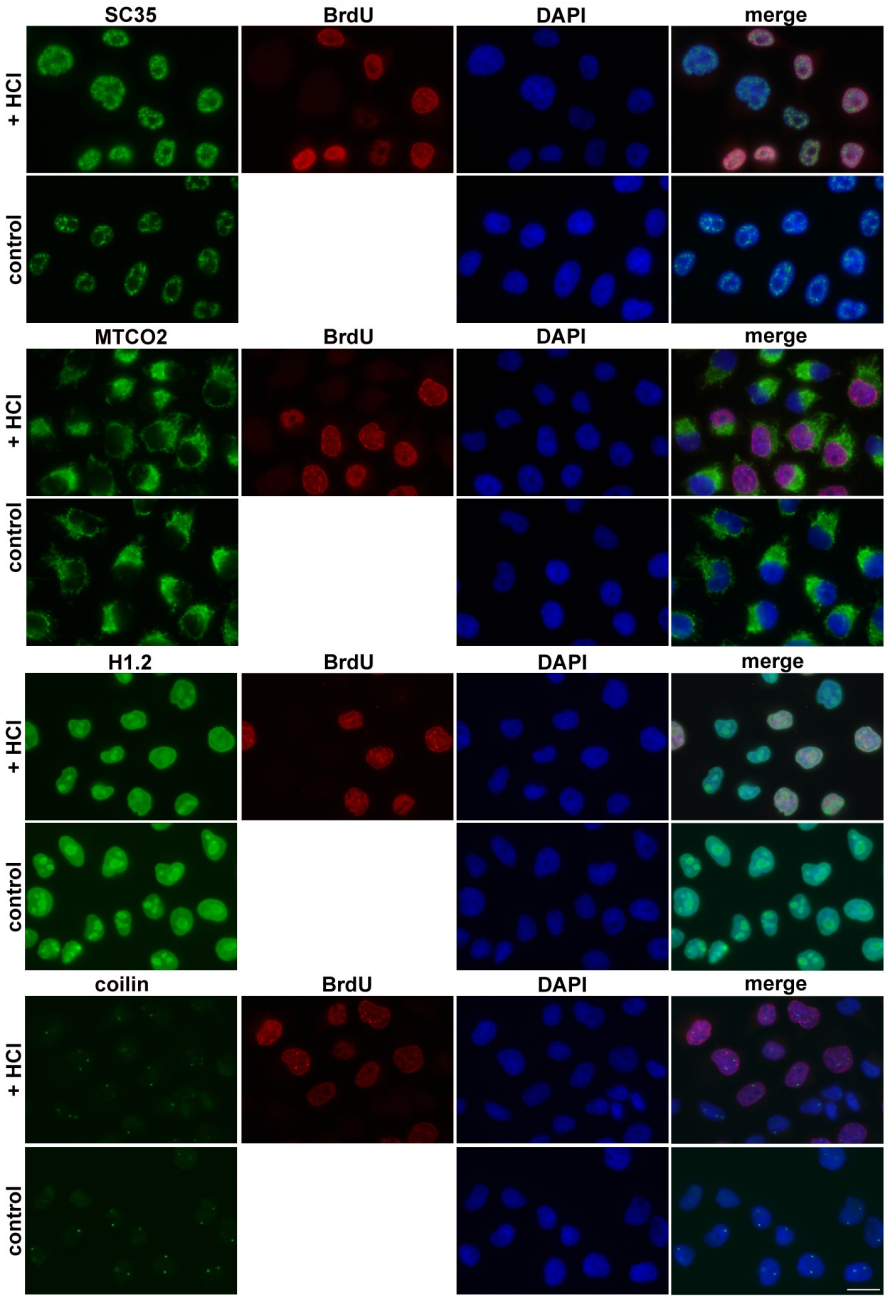
The effect of optimized procedure on the localisation of cellular proteins. HeLa cells were incubated with BrdU for 30 minutes and fixed with formaldehyde. BrdU was revealed using 20 mM HCl. The proteins SC35, mitochondrial protein MTCO2, histone H1.2 and coilin were concurrently detected with the incorporated BrdU. BrdU was detected using either chicken polyclonal antibody or B44 monoclonal antibody depending on the host producing the antibody for the particular cellular protein. The control cells were not labelled with BrdU and were not treated with HCl and exonuclease III. Proteins are in green, BrdU is in red and DAPI is in blue. Scale bar = 20 μm.

The concurrent detection of DNA synthesis and various proteins is a common task in many studies. In this respect, EdU and click chemistry is frequently used. The EdU toxicity is however much higher than the BrdU toxicity. Moreover, the click reaction leads to the formation of oxygen radicals that can negatively influence the sensitive protein epitopes. To determine the impact of 20 mM HCl along with exonuclease III on the detection of various protein components, we analysed the signal and distribution of chosen proteins after the use of the optimised approach. Antibodies raised against the SC35 protein, mitochondrial marker MTCO2 histone H1.2, coilin (Fig 2), NTH1, RPA32, PRAF1, PCNA and MCM7 (S2 Fig) were used. The HeLa cells were incubated with BrdU for 30 min and fixed with formaldehyde. The incorporated BrdU was detected depending on the origin of antibody against analysed proteins either with the B44 clone (if anti-protein antibodies were from rabbit) or with chicken polyclonal antibody (if anti-protein antibodies were from mouse). The results of the analyses showed that the optimized procedure did not exhibit a significant impact on the distribution of analysed proteins (Fig 2, S2 Fig). The intensity of the signal was the same in most cases. Only in the case of PRAF1 did the signal slightly decrease. Taking all these results together and comparing them with our previously published data [7], we can conclude that with respect to the localization of proteins the developed method provides very similar results as the method based on the monovalent copper ions and much better than the protocol based on the use of high concentration of hydrochloric acid.

**Fig 3:**
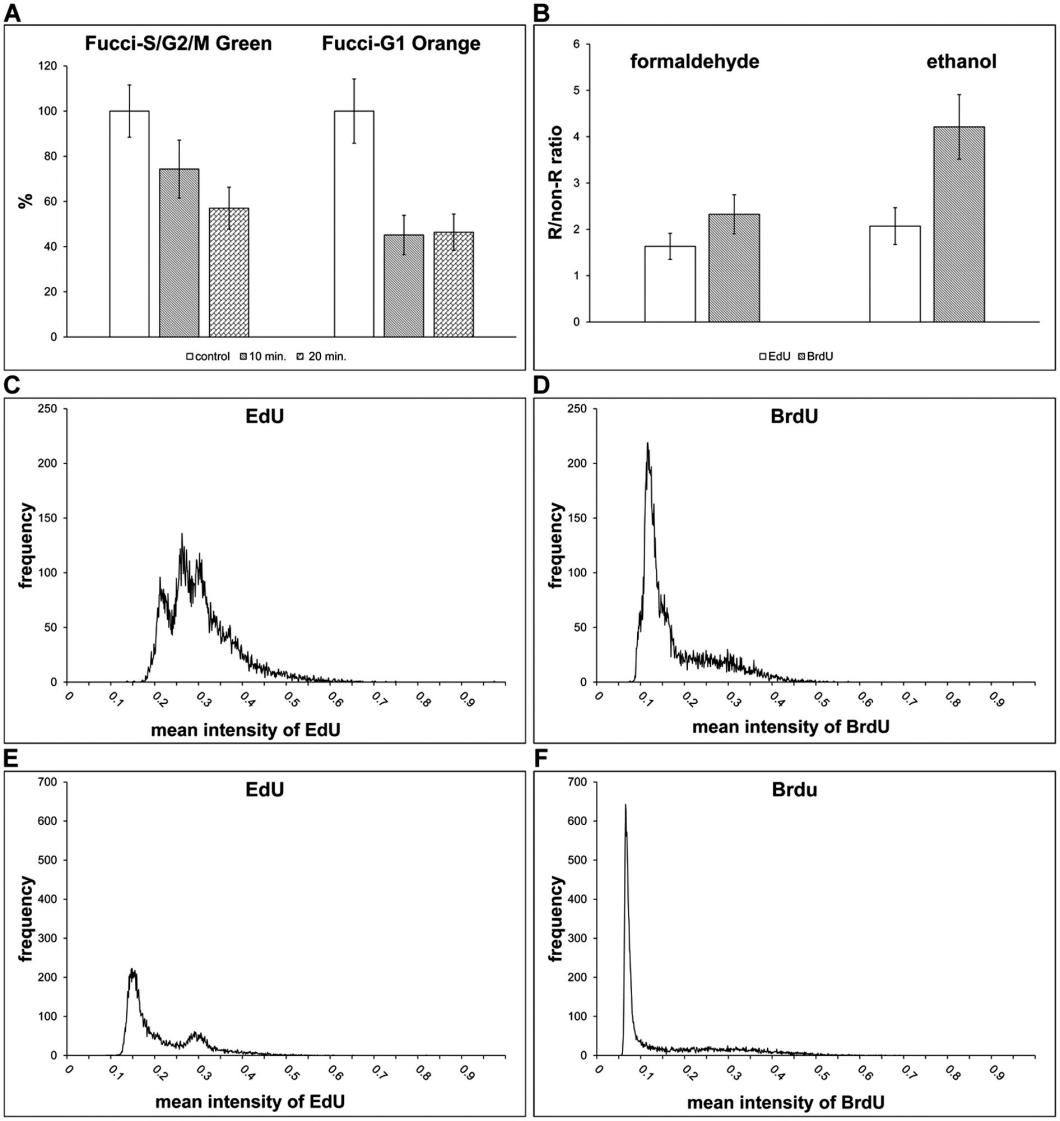
Fluorescent proteins detection and comparison of replicational signal after short pulses of BrdU and EdU. **(A)** The impact of the optimised method on fluorescent proteins is shown. HeLa cells expressing FUCCI were incubated with or without BrdU for 30 minutes and the detection of BrdU using the optimized method was performed. The cells were treated with HCl either for 10 or 20 minutes. The average signal of fluorescent proteins was measured in cells. The data were normalised to the % of the average signal of Fucci-G1 Orange and Fucci-S/G2/M Green in control, non-treated, cells. The data are presented as the mean ± SD. (**B)** The comparison of the signal in cells labelled with 10 μM EdU or BrdU for 5 minutes. The graphs show differences between cells incubated either with EdU or BrdU and fixed either with formaldehyde or ethanol. The data are presented as the mean ± SD. (**C, D)** The histograms of the mean nuclear EdU- (C) or BrdU-derived (D) signals in formaldehyde-fixed cells labelled with 10 μM EdU or BrdU for 5 minutes. (**E, F)** The histograms of the mean nuclear EdU- (E) or BrdU-derived (F) signals in ethanol-fixed cells labelled with 10 μM BrdU or EdU for 5 minutes.

Besides the co-localisation of proteins and DNA replication, the impact of the optimised protocol on the fluorescent proteins in the HeLa cells stably expressing Fucci were tested (Fig 3A). Fucci technology is used for the analysis of cell proliferation using fluorescent probes Fucci-G1 Orange and Fucci-S/G2/M Green [32]. The analysis showed that while the fluorescence of Fucci-S/G2/M Green decreased to ca 60% of the signal measured in control cells, in the case of Fucci-G1 Orange the decrease of the signal was significantly higher (ca 45% of the signal measured in control cells) if samples were incubated in HCl solution for 20 min. The shorter incubation of cells in HCl solution did not result in an increase of the signal of Fucci-G1 Orange, however it resulted in an increase of the signal of Fucci-S/G2/M Green (Fig 3A). In parallel experiments, cells were incubated with EdU and detected by click reaction without the addition of substances used for copper(I) ions stabilization. We observed the complete removal of the fluorescence of fluorescent proteins.

### The approach is suitable for short labelling pulses and for the detection of other modified nucleosides

**Fig 4:**
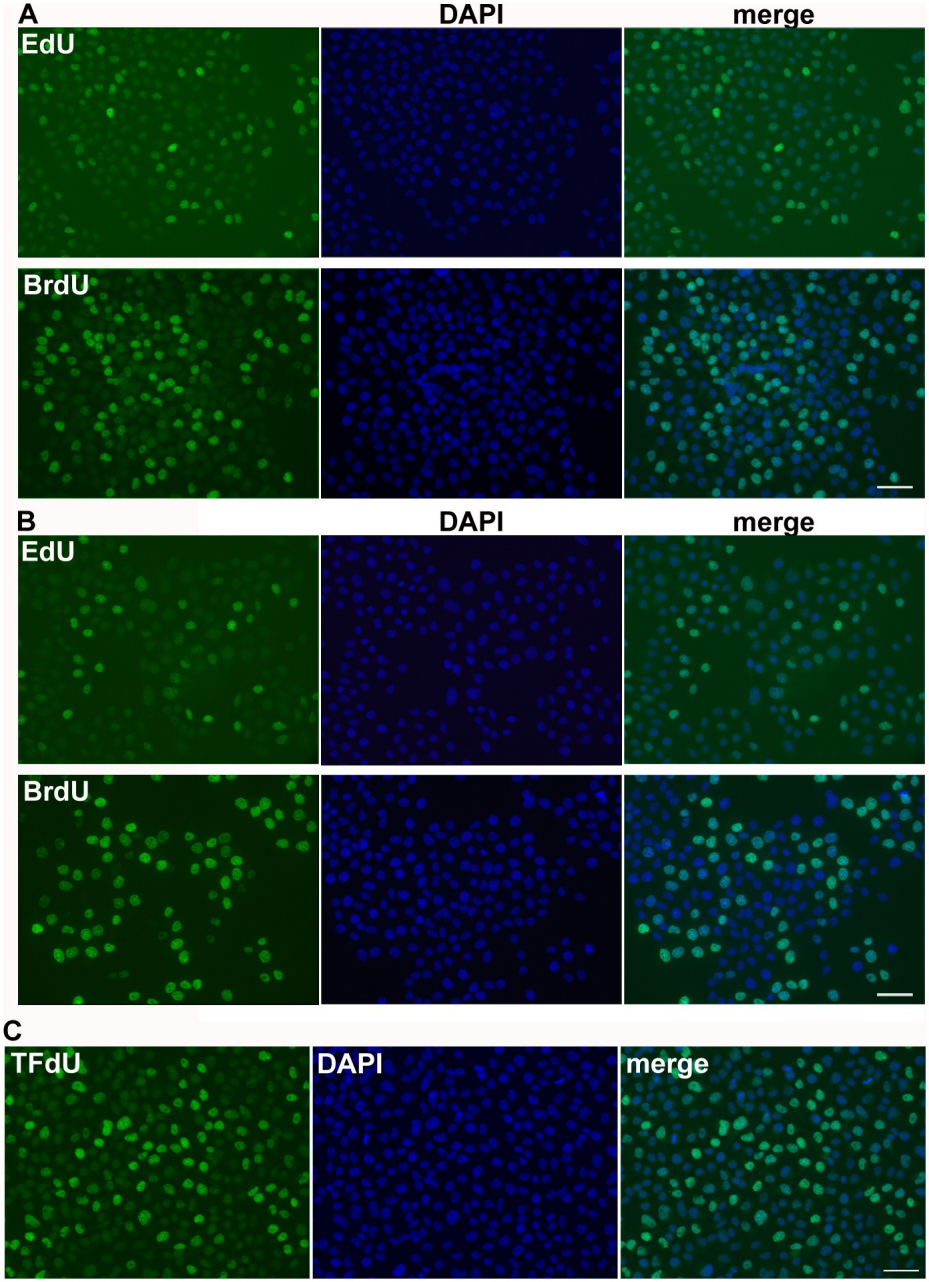
Fluorescence images of BrdU, EdU and TFdU. **(A, B)** The detection of EdU and BrdU in HeLa cells labelled with EdU or BrdU for 5 minutes and fixed with formaldehyde (A) or ethanol (B). EdU and BrdU are in green, DAPI is in blue. The scale bar—50 μm. (**C)** The detection of TFdU. HeLa cells were labelled with TFdU for 30 min. TFdU was revealed by optimized method and detected by anti-BrdU antibody clone 3D4. TFdU is in green, DAPI is in blue. The scale bar—50 μm.

In the studies dealing with e.g. organisation of DNA replication it is often necessary to use short labelling times. Moreover, the short times better reflect the real number of replicating cells. Therefore, we analysed cells after the 5-minute incubation with BrdU or EdU that were fixed either with formaldehyde or ethanol. BrdU was detected using optimised protocol. In formaldehyde-fixed cells, 3D4 antibody was used, in ethanol-fixed cells, Bu20a clone. EdU was detected by click reaction. Our data showed that already a 5-minute incubation with BrdU was sufficient for the detection of replicating cells in the ethanol-fixed cells (Fig 3B). The R/non-R ratio was ca 4 and allowed the reliable differentiation between replicating and non-replicating cells. In the case of EdU, the R/non-R ratio was around 2. According to our results, the ratio around 2 is not sufficient for the reliable differentiation of replicating cells by image and flow cytometry and generally leads to the undervaluation of the number of replicating cells. In the case of formaldehyde-fixed cells, the R/non-R ratio in BrdU-labelled cells was slightly above 2, while in EdU-labelled cells it was under this value (Fig 3B). The inability to use EdU for the reliable identification of replicating cells after short incubation times is also well-documented on the histogram of the average signal in the cell nuclei in formaldehyde-fixed cells (Fig 3C and 3D) and ethanol-fixed cells (Fig 3E and 3F). The difference in the labelling is also obvious from the images obtained from fluorescence microscopy (Fig 4A and 4B).

Next, we tested the optimised method for the detection of TFdU incorporated in DNA. It was shown that this modified nucleoside is incorporated into DNA [28]. In the mentioned study, the authors tested several antibody clones with respect to their ability to detect TFdU. We tested the optimised protocol for the detection of TFdU and found that this protocol enables reliable identification of TFdU-labelled DNA as well (Fig 4C). In agreement with the published results [28], we found that the 3D4 clone is the most suitable clone for TFdU detection.

### The optimised protocol is suitable for flow and image cytometry

**Fig 5:**
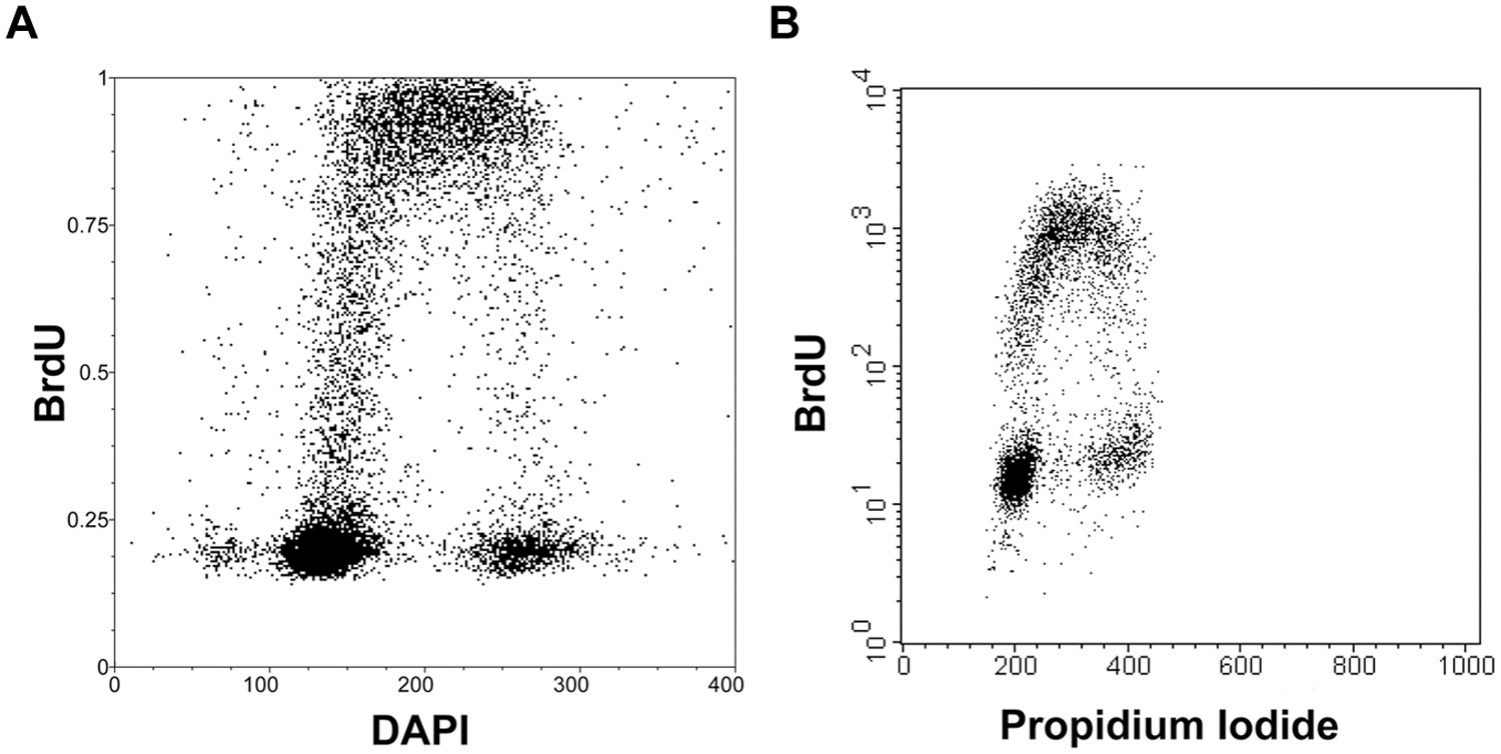
Determination of cell cycle by image and flow cytometry. **(A)** The bivariate analysis of the replication signal and DNA content by DAPI in HeLa cells after a 30-minute incubation with 10 μM BrdU using image cytometry. (**B)** The bivariate analysis of the replication signal and DNA content by propidium iodide in A549 cells after a 30-minute incubation with 10 μM BrdU using flow cytometry.

Finally, the optimised method for the determination of cell cycle profile was tested using image cytometry (Fig 5A) and flow cytometry (Fig 5B). In the case of flow cytometry, the best option was found to be the protocol based on the ethanol fixation. This protocol led to the minimal loss of cells during the samples processing and concurrently provided the high signal/background ratio. In contrast, both fixation methods were suitable for image cytometry applications. Moreover, the use of a low concentration of HCl and exonuclease III for the detection of BrdU has no effect on the DNA content when compared to the control cells (S3 Fig.).

The optimised protocols for image and flow cytometry are described below:

#### Image cytometry

**1**. Wash with 1× PBS **2**. Fixation

**Formaldehyde fixation:** Fix the samples with 1–2% formaldehyde in 1× PBS (10 min, RT), wash with 1× PBS (3×) and permeabilise with 0.2% Triton X-100 (10 min, RT). Then, wash the samples with 1× PBS (1 hr in total, RT). Generally, a longer wash step is not critical, but its shortening leads to the decrease of the signal.

**Ethanol fixation:** Fix the samples with 70% ethanol (30 min-several weeks, -20°C)

1. BrdU detection
  - Wash with 150 mM NaCl and 3 mM KCl (3x). This step serves for washing out 1× PBS. The residual 1× PBS neutralises the acid environment in the next step leading to a decrease of the signal.
  - Incubate in the solution of 5–20 mM HCl in 150 mM NaCl and 3 mM KCl (20 min, 25°C). Although the higher temperature leads to the increase of the number of DNA breaks, it simultaneously leads to an increase of the cell structure damage. On the other hand, the lower temperature requires the prolongation of the incubation time and/or higher concentration of acid for sufficient BrdU detection. The concentration of HCl depends on the fixation protocol and antibody used. Test it at first. According to our data, 20 mM HCl will usually work well.
  - Wash with 1× buffer for exonuclease III (3x). This step serves for the neutralisation of the acidic environment. According to our observations, one wash step is enough.
  - Incubate the samples in a mixture of primary anti-BrdU antibody (for the B44clone the optimal concentration is 0.25–0.5 μg/ml, for Bu20a 2.5–5 μg/ml, for other clones test the optimal concentration), exonuclease III (0.4 U/μl) and 1× buffer for exonuclease III (30 min, 37°C). If it is necessary to incubate the samples at RT, increase the exonuclease III concentration twice. If the RNA has to be removed, it is possible to add to the mixture RNase A (100 μg/ml). Concerning the primary antibodies, there are many anti-BrdU clones available. The guide for the selection of antibody from antibodies tested in this study is Fig 2E–2F. Generally, the higher R/non-R ratio is more advantageous than the lower one. A ratio higher than 3 enables reliable identification of replicating cells. The values in the graph were measured in HeLa cells incubated with 10 μM BrdU for 30 min. In the case of shorter incubation times, the ratio will be lower and the selection of the antibody with the highest R/non-R ratio may be a critical for the faithful determination of replicating cells.
  - Short wash with 1× PBS (2x, in total use the washing time is 1 min or less). The quick wash followed by stabilisation step is important in ethanol-fixed cells only for some antibodies. On the contrary, for formaldehyde-fixed cells, this step is important for the majority of the antibodies tested (Fig 1F & 1G). It is evident from our unpublished data that the stabilisation of the primary antibody can be also achieved by immediate incubation with the secondary antibody. Therefore, if the stabilisation step is not used, a good signal can be obtained by the quick reaction with the secondary antibody although the stabilisation step usually allows faster and more effective stabilisation of the antibody.
  - Incubate with 0.2% formaldehyde in 1× PBS (10 min, RT). It is possible to use a methanol-stabilised stock solution of formaldehyde or solution prepared from paraformaldehyde in 1× PBS.
  - Wash with 25 mM Tris-HCl, pH = 7.5 and 150 mM NaCl (3x 5 min). It is possible to perform only one wash without significant effect on the secondary antibody reaction.
  - Incubate with the secondary antibody and DAPI (10 μM) in 25 mM Tris-HCl, pH = 7.5 and 150 mM NaCl (30 min, RT).
  - Wash in 25 mM Tris-HCl, pH = 7.5 and 150 mM NaCl (3x 5 min).

#### Flow cytometry

As it involves very similar steps as the protocol for image cytometry for common notes on individual steps, see the protocol for image cytometry.

1. Incubate the samples with 10 μM BrdU (30 min).
2. Centrifuge the samples (500x *g*, 5 min).
3. Discard the supernatant and add 1× PBS (10 ml if 15 ml tubes are used).
4. Centrifuge the samples (500x *g*, 5 min).
5. Discard the supernatant and add 150 mM NaCl a 3 mM KCl. Mix by pipetting.
6. Slowly drop 7 ml of 100% ethanol cooled on -20°C. Put the samples into the freezer and incubate at least for 60 min. It is possible to leave the samples in the freezer for several weeks without loss of the signal. A shorter time did not result in a decrease of the signal, however it can result in the gradual loss of cells.
7. Centrifuge the samples (500x *g*, 5 min).
8. Discard the supernatant and add 3 ml of 10–20 mM HCl in 150 mM NaCl and 3 mM KCl. Mix by pipetting (10x) and incubate for 20 min, 25°C. During incubation gently shake the tube, do not vortex.
9. Add 0.3 ml of 10× buffer for exonuclease III, mix by pipetting (it neutralises the acidic solution).
10. Centrifuge the samples (500x g, 5 min).
11. Discard the supernatant and add 150–200 μl of the mixture composed of exonuclease III (0.4 U/μl), RNase A (100 μg/ml) and primary antibody (Bu20a, 0.5 μg/ml) in 1× buffer for exonuclease III (30 min, 37°C). During incubation gently shake the tube, do not vortex.
12. Add 3 ml of 1× PBS, mix by pipetting and immediately add 15 μl of 35% stabilised formaldehyde (5 min, RT). This step is important when low stability of the particular antibody clone/BrdU complex is expected due to the relatively long time necessary for centrifugation. In other cases, this step can be skipped. However, the time of centrifugation and washing should be minimised.
13. Centrifuge the samples (500x *g*, 10 min).
14. Discard the supernatant and add 150 μl of the mixture of the secondary antibody, propidium iodide (10 μg/ml) in 25 mM Tris-HCl, pH = 7.5 and 150 mM NaCl. Mix by pipetting (10x) and incubate 30 min, RT.
15. Centrifuge the samples (500x *g*, 10 min).
16. Discard the supernatant and add 5 ml of 1× PBS. Mix by pipetting.

## Conclusions

The aim of this study was the development of a method enabling the highly reliable detection of replicational activity using BrdU both in adherent and suspension cell cultures by image and flow cytometry. The method enables a high degree of cell structure preservation. Although the developed approach is based on the hydrochloric acid application, its concentrations are compared to the commonly used ones 75–800 times lower. Therefore, it is not surprising that the performed analyses of the chosen proteins with respect to their distribution and signal strength showed that the presented technology allows the concurrent analysis of the whole range of proteins. In addition, it also enables at least to some extent the performance of co-localization studies with fluorescent proteins. As the immunocytochemical detection of proteins can vary from protein to protein and from antibody to antibody, the impact of the method on the localization and signal intensity of particular protein should be tested first.

In agreement with our previous results, we showed that the level of the specific signal is dependent on the antibody used [24]. Our findings are also in agreement with our non-published results showing the need of stabilisation of some anti-BrdU antibodies. For their stabilisation, a short incubation with formaldehyde is sufficient. This step was essential only in the case of some antibodies. However, e.g. in the case of the clone Bu20a, the stabilisation step led to such a high increase of the specific signal that this anti-BrdU clone can be recommended for the majority of the analyses with ethanol-fixed cells. In the case of formaldehyde-fixed cells, the clone B44 or 3D4 is the more suitable choice.

According to our results the ethanol fixation provides a commonly higher signal and is therefore recommended in all situations where the signal intensity plays the most important role. In addition, this fixation enables the lowering of anti-BrdU antibody concentrations at least in the case of some antibodies. For instance, the five times lower concentration of the B44 clone in ethanol-fixed cells was sufficient to achieve the same signal strength as in formaldehyde-fixed cells.

We also compared the results with the results of the method based on the use of EdU. Contrary to the approach based on the use of EdU, the described optimised method is much more suitable for the short pulses with the nucleoside marker. As this method enables also detection of TFdU and considering the fact that the described approach does not exhibit a strong impact on the cellular proteins, it is possible to use it in experiments focused on the principle of the effect of TFdU and the derived drug TAS-102 on cells.

## Supporting information

S1 Fig Comparison of the R/non-R ratio using various methods of BrdU detection. HeLa cells were incubated with BrdU for 30 minutes and fixed with formaldehyde. BrdU was revealed using 20 mM HCl and exonuclease III or 2N HCl or 4N HCl or monovalent copper ions or DNase I (20U/ml). The incorporated BrdU was detected by anti-BrdU antibody clone Bu20a. The data are presented as the mean ± SD.
(TIF)

S2 Fig The effect of the optimized procedure on the localisation of cellular proteins. HeLa cells were incubated with BrdU for 30 minutes and fixed with formaldehyde. BrdU was revealed by 20 mM HCl and exonuclease III treatment. The proteins NTH1, RPA32, PRAF1, MCM7 and PCNA were concurrently detected with the incorporated BrdU. The control cells were not labelled with BrdU and were not treated with HCl and exonuclease III. Proteins are in green, BrdU is in red and DAPI is in blue. Scale bar = 20 μm.
(TIF)

S3 Fig Comparison of DNA content in control and HCl-treated cells. HeLa cells were incubated with BrdU for 30 minutes and fixed with formaldehyde. BrdU was revealed using 40 mM HCl and exonuclease III. The control cells were not labelled with BrdU and were not treated with HCl and exonuclease III.
(TIF)
