# Supplementary figures and images for: Cell cycle profiling by image and flow cytometry: The optimised protocol for the detection of replicational activity using 5-Bromo-2′-deoxyuridine, low concentration of hydrochloric acid and exonuclease III
Source: PLoS One. 2017 Apr 20;12(4):e0175880. doi: 10.1371/journal.pone.0175880 (PMC5398562; doi:10.1371/journal.pone.0175880)

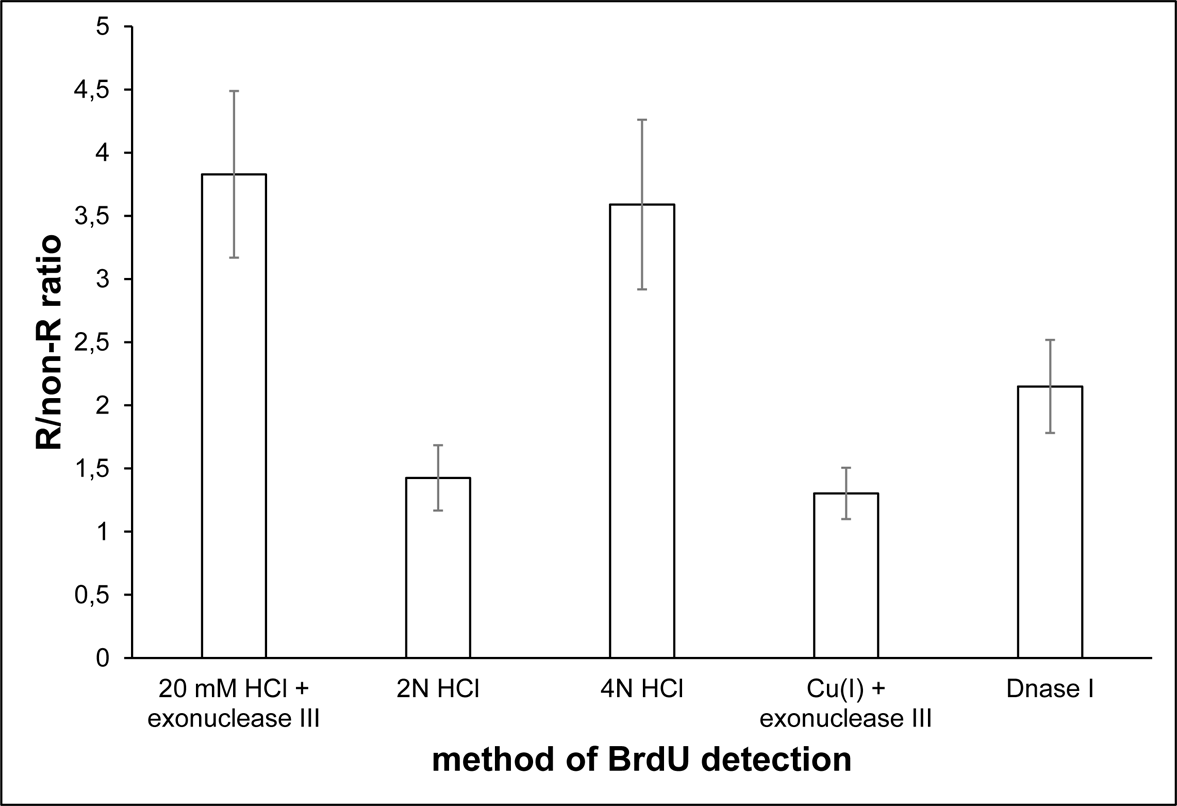

Supplement: S1 Fig — HeLa cells were incubated with BrdU for 30 minutes and fixed with formaldehyde. BrdU was revealed using 20 mM HCl and exonuclease III or 2N HCl or 4N HCl or monovalent copper ions or DNase I (20U/ml). The incorporated BrdU was detected by anti-BrdU antibody clone Bu20a. The data are presented as the mean ± SD. (TIF) [file pone.0175880.s001.tif]

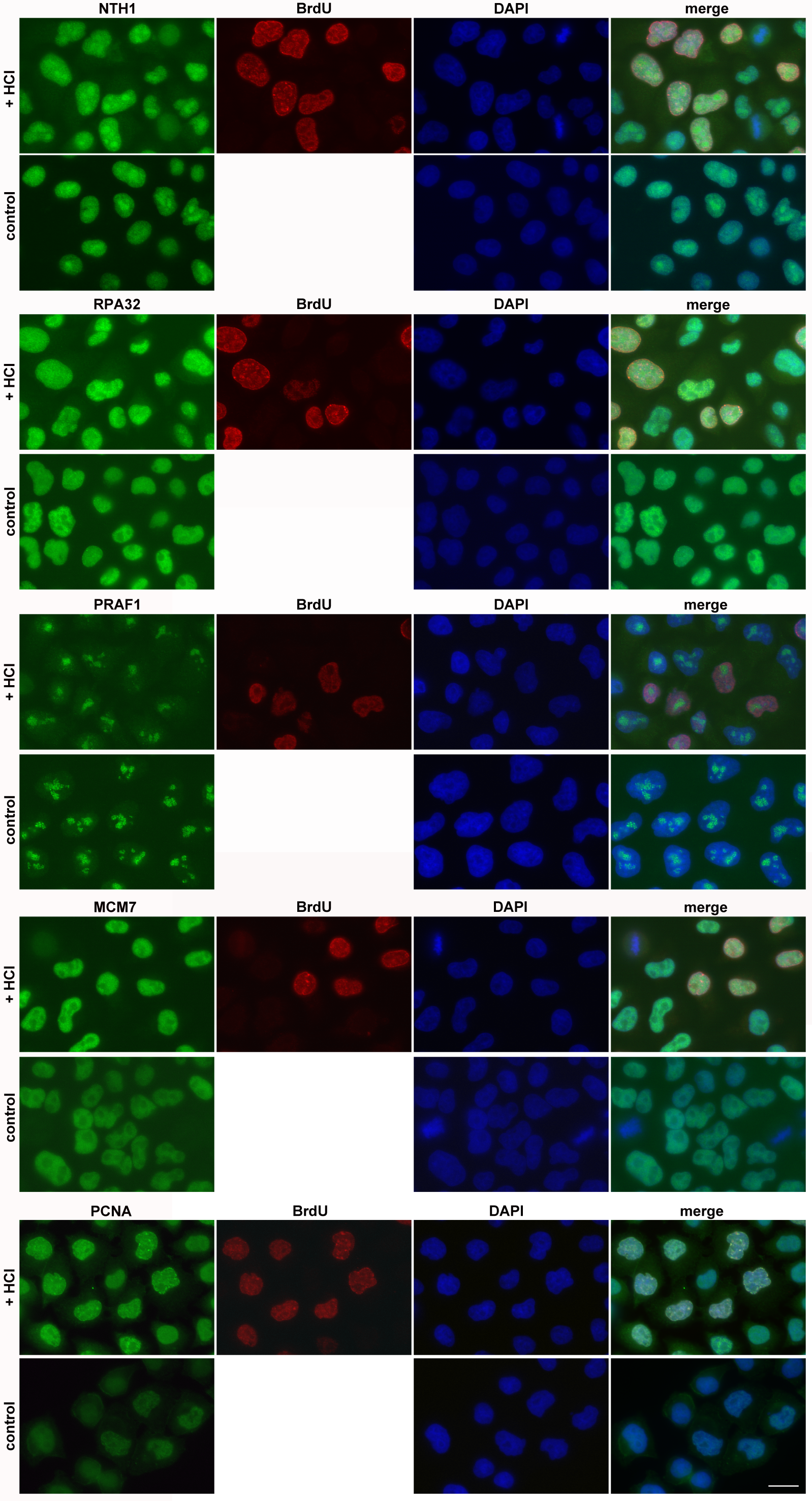

Supplement: S2 Fig — HeLa cells were incubated with BrdU for 30 minutes and fixed with formaldehyde. BrdU was revealed by 20 mM HCl and exonuclease III treatment. The proteins NTH1, RPA32, PRAF1, MCM7 and PCNA were concurrently detected with the incorporated BrdU. The control cells were not labelled with BrdU and were not treated with HCl and exonuclease III. Proteins are in green, BrdU is in red and DAPI is in blue. Scale bar = 20 μm. (TIF) [file pone.0175880.s002.TIF]

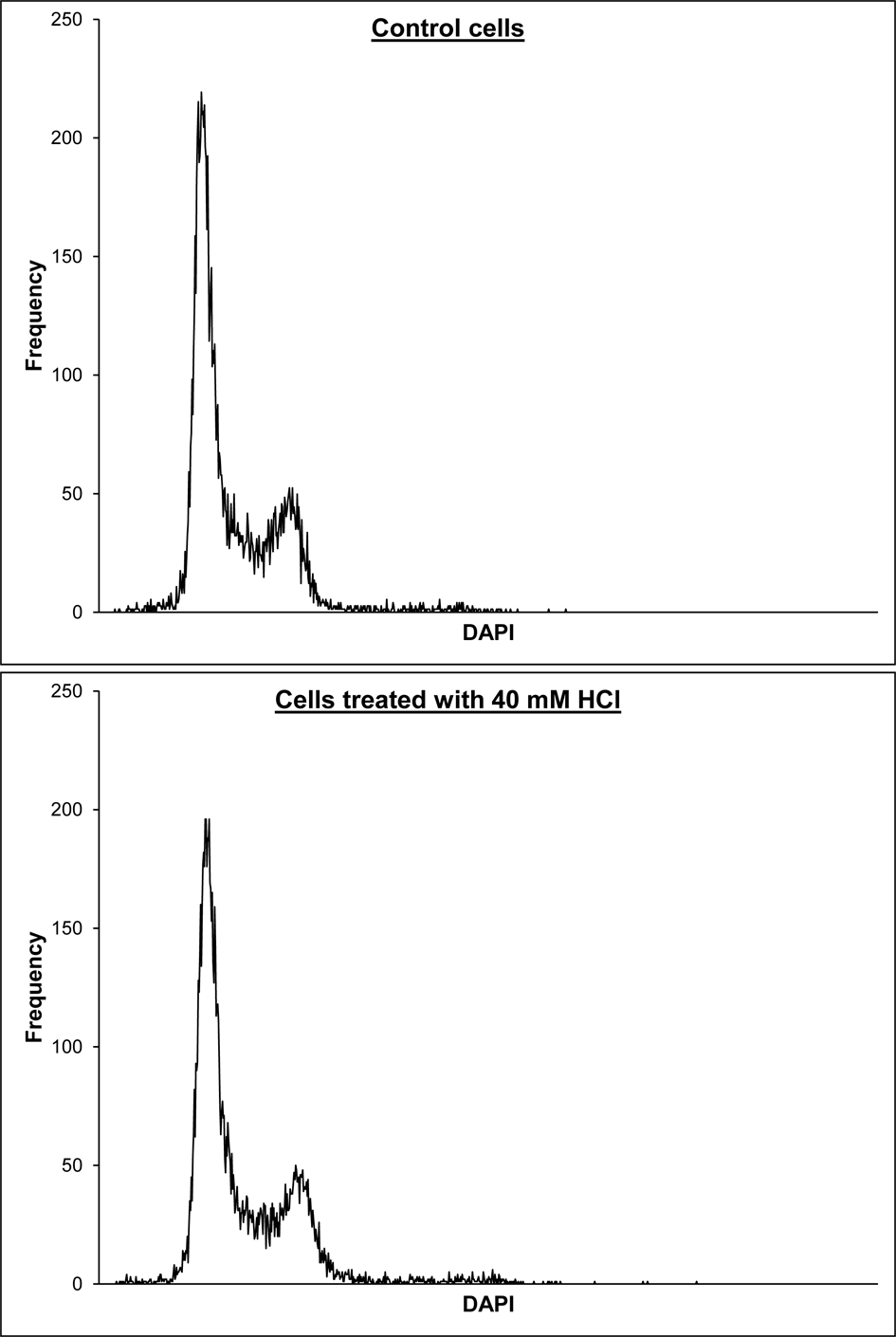

Supplement: S3 Fig — HeLa cells were incubated with BrdU for 30 minutes and fixed with formaldehyde. BrdU was revealed using 40 mM HCl and exonuclease III. The control cells were not labelled with BrdU and were not treated with HCl and exonuclease III. (TIF) [file pone.0175880.s003.tif]
